# Integrated secure distance bounding and hardware-based security: A case study for the insurance claim verification of farmers during COVID-19

**DOI:** 10.12688/openreseurope.15448.1

**Published:** 2023-02-23

**Authors:** Alper Kanak, Salih Ergün, İbrahim Arif, Sercan Tanrıseven, Niyazi Uğur, Gert-Jan van Schaik, Atta Badii

**Affiliations:** 1 Research and Development, ERARGE, Ergünler Co. Ltd., Isparta, 32100, Turkey; 2 Research Center, ERGTECH, Zurich, Switzerland; 3 Stichting IMEC, Eindhoven, Netherlands Antilles; 4 University of Reading, Reading, UK

**Keywords:** hardware-based security, authentication, Internet of Things, X-as-a-Service, secure distance bounding, cryptography, blockchain, proximal distance verification

## Abstract

Given the rapidly evolving developments in Fintech, Insurtech, Open Banking, and Mobile Money business models in recent years, the capability for ensuring strong authentication remains the most pressing need for the protection of security and privacy of data in this sector as in many other areas. The security-integrity of insurance and financial transactions and workflows is vitally dependent on access control mechanisms to deliver strong multi-factor authentication (MFA) with operationally acceptable latency and throughput to support real-time response, particularly as demanded by the increasing online and mobile financial service models.

The Critical-Chains Project was motivated by the above objectives as underpinned by the overarching commitment to accountability engineering as required by the operational logic. This must be crucially supported by real-time hardware-enabled services comprising authentication (including Distance Bounding and Prover’s Proximal Location Presence Verification), hardware security and cryptography (AUTH-as-a-Service, Hardware-Security-as-a-Service, Cryptography-as-a-Service) as delivered through the Critical-Chains main framework.

This paper reports on the development and evaluation of the innovative Hardware-enabled authentication and security capabilities of the Critical-Chains framework which is successfully validated in the context of financial services, specifically the insurance claim settlement application domain, and can also be deployed in any other domains where trusted authentication and specific location-time bound prover’s presence verification is required.

## Introduction

Fundamentally, secure access control is predicated on the availability of secure authentication processes which are in turn enabled by strong cryptographic solutions including truly random number generation-based protocols as non-deterministic processes based on a dynamic and stable source of entropy.

On the other hand, the scalability of such secure authentication solutions requires acceptable latency and throughput for the requisite cryptographic processes, and this points to a hardware-enabled solution as the platform for delivering strong authentication.

However further security safeguarding measures are required to prevent impersonation and man-in-the-middle attacks, particularly in applications, such as in keyless access in the automotive sector, where the authentication and verification of time-limited location proximity of the Prover’s Presence, as well as anti-tampering, are required to enable protection against impersonation and relay type (man-in-the-middle) cyber-attacks. Additionally, the accountability and integrity of the back-end database have to be assured as can be supported through integrating a Blockchain-as-a-Service layer. This paper describes the architecture, implementation, and validation of the solution stack developed and tested, applied to the insurance claim settlement domain, under the Critical-Chains project. The validation results show that the above challenges have been successfully addressed to arrive at a robust and innovative solution stack comprising a Cyber-Physical Security-as-a-Service (CPSaaS) framework providing integrated authentication and cyber-resilience through cryptographic and Blockchain capabilities.

## 1 State-of-the-Art Update

The scope of the paper is broad as the presented work has a strong background that is based on the current state of the art. Although the main objective of the authors is to establish the framework motivated by and validated in the Critical-Chains project using a realistic use-case and shed light on potential and practical uses of the developed solution stack, the presented technologies can be used as a scientific reference by the related research and industry stakeholders. Aligned with this goal, this section presents an overview of the literature in the main focus areas of relevant research, such as authentication mechanisms, secure and Internet of Things (IoT)-enabled blockchain frameworks and their uses in the finance and insurance sectors.

### 1.1 Person and IoT nodes authentication and their uses in Fintech and Insurtech

The recent state-of-the-art for authentication mechanisms has evolved from person authentication to node authentication. Internet-based services have advanced fast in the last decade as IoT-based solutions diversify and become widespread. Aligned with this trend, open-source protocols, and services such as Message Queuing Telemetry Transport (MQTT) and Constrained Application Protocol (CoAP), and recently LinkSmart, rely on open standard authorisation protocols such as OAuth ^1^.

The majority of the solutions focus on utilising approval tokens to demonstrate an identity among consumers and services rather than sharing secret key information. For instance, OAuth is an authentication protocol that enables a user to support an application interfacing with another for their benefit without endlessly giving the password. In the colossal-scale IoT network, which is connected with huge numbers of sensors and other devices, identifying one component raises a fundamental challenge, because it could cause issues regarding privacy protection governance, access control, and overall architecture. A recent review paper ^2^ presents an overview of three security requirements of an IoT-enabled cyber-physical system: confidentiality, integrity, and availability. The environment of IoT may differ from a centralised network to a decentralised network or a cloud-to-fog network. Therefore, security can be further tightened by enforcing techniques for the detection of unusual behaviour or pattern of the network.

In recent years, authentication over decentralised networks has advanced. In a recent study, authors presented the BCTrust which is implemented over Ethereum Blockchain and IoT for devices with computational, storage, and energy consumption constraints ^3^. Mohanta *et al.,* presented DecAuth ^4^ for multipurpose heterogeneous IoT platforms, again based on the Ethereum blockchain. Blockchain-enabled authentication mechanisms have also been applied to the finance sector. For instance, Xenya and Quist-Aphetsi proposed an application of a blockchain to financial transaction backup data ^5^. By using a decentralised distributed blockchain ledger, each node can have a copy of the transaction data such that, failure in one node would not risk a total failure in transaction data. In another study Kabra *et al.,* presented MudraChain ^6^ as a blockchain-based framework for automated cheque clearance in financial institutions. Very similar to Critical-Chains, the authors presented a two-factor authentication protocol to generate a time-based One-Time Password (TOTP) for secure funds transfer.

Biometric authentication has been studied in many areas including the finance sector ^7^. Biometrics have been widely used in Point of Sale (POS) networks ^8^ where fingerprints, palm, and finger vein biometrics and facial biometrics are used. There also exist new approaches whereby multimodal biometrics are deployed because unimodal techniques that rely on single biometric modalities, fingerprint-only, face-only, or iris-only solutions may have specific in terms of accuracy, practicality, or cost-effectiveness ^9^. Another study presented a conceptual framework using multimodal biometrics in financial risk prevention and control, e.g., big data credit reporting. An interesting study proposed a biometric currency concept that enabled people to self-finance and safely store their money under their control (so that can be issued not only by bankers but also everyone) ^10^. Biometrics have applications to also in blockchain technology. For example, Páez *et al.,* proposed an architecture for a biometric electronic identification document (e-ID) system based on Blockchain for the citizens’ identity verification in transactions corresponding to the notary, registration, tax declaration and payment, basic health services and registration of economic activities ^11^.

Blockchain-enabled authentication mechanisms have been applied in the insurance sector. For instance, Xiao *et.al.,* presented a trustable blockchain-enabled transaction authentication method that utilised homomorphic encryption. This approach has been adapted to several variants of insurance data security transaction authentication ^12, 13^. Amponsah *et.al.,* presented useful architectures for insurance claim submission and processing over decentralized networks, fraud detection during claim submission or policy issuance, and Know-Your-Customer (KYC) compliance using blockchain ^14^. Recent studies show that blockchain-enabled contracts are usually integrated with either very basic tokens or large but cumbersome databases. There is a strong need to integrate IoT-enabled sensory systems in decentralised databases dealing with real-time or near-real-time services ^15^. A recent survey addressed the current state of play and strategies for the transition towards more IoT-enabled and rapidly–responsive blockchain infrastructures in the Fintech and Insurtech domain ^16^.

### 1.2 Hardware-based Cyber-Physical Security

Financial cryptography, or cryptography in Finance including both Fintech and Insurtech, is not a new concept but has been considered for centuries since the first days of the invention of money. Financial cryptography is a substantially complex topic which requires comprehensive and elaborated security schemes, not only covering transaction security but also privacy preservation both at an individual and organisational level. Financial cryptography has become a broad scientific research area that incorporates many disciplines such as accountancy and auditing, programming, system-of-systems, economics, Internet, finance and banking, risk management, marketing and distribution, central banking, and recently, in the last decade, hardware-based cyber-physical security, AI-powered security, and their uses in decentralised blockchain environments ^15, 16^.

Cyber-physical security is a very wide topic as it has numerous applications in all domains not only Fintech and Insurtech but also in the automotive domain, health, Industry 4.0, aerospace, space, transportation, smart cities, etc. In this study, we mainly focused on the use of hardware-based IoT-enabled cryptographic solutions that are used for collecting and transmitting critical financial and insurance-related data over decentralised networks. Recent trends have shown that hardware-based cryptographic solutions have become indispensable. The token-based authentication systems are still dominant, especially for mission-critical approaches. Token-based authentication techniques are incorporated with the advanced cryptographic hardware, for example, HSMs, on the server side. The FIDO standard also supports the easy use of token-based authentication, especially for person authentication ^17^. Beyond person authentication, authentication of nodes, or in general things (e.g., IoT), has been evolving. For instance, Dammak *et.al.,* ^18^ presented a token-based lightweight authentication scheme for IoT networks which generates an additional security layer by adopting the token technique offering access to a specific resource within a period. Karim *et. al.,* ^19^ presented a digital signature authentication for a bank using asymmetric key cryptography and token-based authentication by an OTP mechanism. In a similar study, the authors presented security services including X.509 certificate, RSA-based Public Key Infrastructure (PKI), and challenge/response protocols with the help of a proxy-induced security service provider ^20^.

IoT, token-based authentication, and blockchain have become complementary areas that are bridged in new-generation security schemes. For instance, Park *et.al.,* ^21^ focused on the certification technology suitable for small-scale IoT environments and proposed a system in which many gateways share authentication information and issue authentication tokens for mutual authentication using blockchain. Hardware-based security covers a broad range of topics from trusted computing to Trojan circuits. Among these, secure platforms are accepted as the Root of Trust, providing security functionality. At this high level of abstraction, the system designer receives a complete chip or board as a trusted computing base. The system designer assumes that the trusted root delivers a set of cryptographic functions, protected by the hardware and software inside the physical enclosure. Common to these platforms is that they are stand-alone pieces of silicon with a strict access policy. Depending on the provided functionality, the hardware tamper resistance and protection levels, and the communication interface, these secure platforms are used in different application fields (automotive, financial, telecom). The three most important platforms are the Hardware Security Module (HSM), the Subscriber Identification Module (SIM) and the Trusted Platform Module (TPM) ^22^.

HSMs play a crucial role in secure platforms which typically provide cryptographic operations, for example, a set of public key and secret key algorithms, together with secure key management including secure generation, storage, and deletion of keys. Essential to HSMs is that these operations occur in a hardened and tamper-resistant environment. A True Random Number Generator (TRNG) and a notion of a real-time clock are usually included. HSMs are mostly used in server back-end systems to manage keys or payment systems, for instance, in banking systems. Security and privacy rely on strong cryptographic algorithms and protocols and random number generation which plays a crucial role to enable unpredictability and non-determinism. A dynamic and stable source of entropy is essential in these protocols: random numbers are used to generate session keys, nonce, initialisation vectors, to introduce freshness, etc. Random numbers are also used to create masks in masking countermeasures, random shares in multi-party computation, zero-knowledge proofs, etc. Pseudo-random number generators are widely used especially at the software level, but they rely on deterministic algorithms that generate a sequence of bits or numbers that look random but are generated by a deterministic process. However, TRNGs rely on a hardware-based entropy source, which is a physical phenomenon with random behaviour. In electronic circuits, noise or entropy sources are usually based on thermal noise, jitter, and metastability. The foremost techniques in advanced TRNG design have adapted the operation of continuous-time chaos ^23^, discrete-time chaos ^24^, ring oscillators ^25^, tetrahedral oscillators ^26^, or other nonlinear techniques as an entropy source to generate truly random numbers.

The state of the art in distance bounding is finding much interest as a means of authentication supported by time and location proximal presence verification. Distance bounding can be realised by relying on various communication protocols, such as Near Field Communication (NFC), ultra-wideband (UWB), WiFi, Radio Frequency Identification (RFID), low-frequency devices, and bluetooth ^27^. UWB solutions show some promise, but the technology is not yet widely used. Bluetooth, on the other hand, is already supported by a vast commercial ecosystem and is often built into smart devices.

The distance bounding technologies are not fully resilient against cyber-attacks. One of the major challenges to be overcome is the vulnerabilities due to the relay attacks or relay station attacks ^28^. Such relay stations are not necessarily restricted by the same communication range limits as legitimate entities. This gives relay stations the ability to simply decrease the measured proximity between two legitimate entities by relaying their mutual communications. Low-frequency devices and systems using a Received Signal Strength Indicator (RSSI) for distance bounding are the most vulnerable attack surfaces.

A relay attack or relay station attack does not require any knowledge of the actual data that is being transmitted, which means you cannot protect the data by using cryptographic measures. The way to effectively mitigate such relay attacks is by implementing secure distance bounding (SDB) protocols which are ambitiously addressed in the Critical-Chains project. In the automotive sector, today, keyless entry systems are based on low frequency (LF) radio technology which is both impossible to support on smart devices (phones, watches, etc.) as well as insecure against wireless relay attacks. Hence, technologies already in smart devices, such as Bluetooth are highly relevant to the attainable systemic security overall. Large industry consortia such as Car Connectivity Consortium (CCC) with its Digital Key standardisation effort as well as the Fine Ranging Consortium have brought together OEM, Tier1, and Tier2 companies to standardise secure distance bounding applications using next-generation wireless technologies. With its very large industry footprint, bluetooth is poised to take a leading position for Secure Distance Bounding (SDB) applications, besides the more power-consuming and costly UWB solutions ^29^.

## 2 Critical-Chains solution

The overall Critical-Chains architecture is based on several main ’components’ that are implemented by considering a service-oriented architecture. The main framework enables the handling of data comprehensively, by managing data storage and injection, streaming and notification services as well as the search and visualisation capabilities. This core foundation enables the solution to handle all the possible use cases that may require deep data integration and transition.

All the layers that enable application user interfaces to execute business logic in a secure and structured way are built on top of the framework. A Cyber-Physical Security-as-a-Service (CPSaaS) layer provides main authentication, authorisation, cyber-resilience, and cryptographic and Blockchain capabilities. All such capabilities are featured as services, in the form of X-as-a-Service. The Blockchain-as-a-Service (BCaaS) is implemented through a resilient keyless signature infrastructure and an Ethereum-based private Blockchain supporting the deployment of smart contracts for both case-specific applications and the authorisation of stakeholders. Authentication and cryptography features are provided through a strong Single Sign On (SSO) architecture as well as hashing and physical hardware devices for user authentication, namely Authentication-as-a-Service (AuthaaS), Hardware Security-as-a-Service (HwSaaS), and Cryptography-as-a-Service (CryptaaS). The flow modelling component, namely Flow Modelling-as-a-Service (FMaaS) is based on adapted artificial intelligence (AI) and machine learning (ML) algorithms that provide financial anomaly detection and transaction flow analytics to detect fraudulent financial transactions. The main framework is protected by a secure cyber Framework that provides security threat detection capabilities and preventive measures against network intrusions and cyber-physical attacks on the main framework.

**Figure 1.**
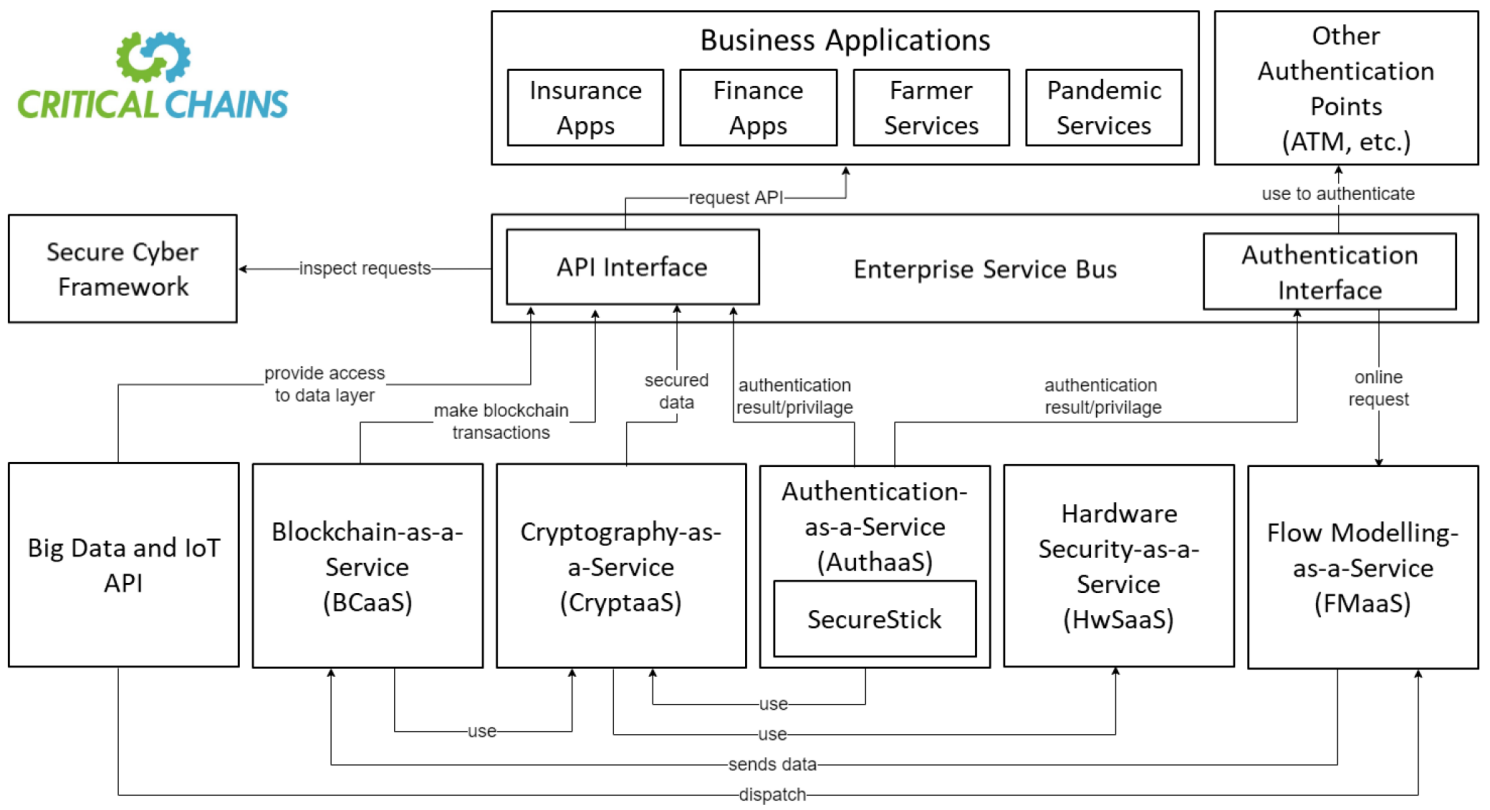
Critical-Chains Main Framework Architecture.

Additionally, the building blocks include several components to support the operation of this innovative framework and are depicted in Figure 1. An open-source enterprise service-bus component provides the orchestration of all the API calls to the CPSaaS and Critical-Chains main framework components and enables eff queue management in order not to eliminate the risk of losing any transactions as well as supporting the core services such as Blockchain, cryptographic functions, financial flow analysis, and authentication.

BCaaS enables the triggering of smart contract calls to generate secure transactions as well as using an electronic signature module to enable transaction signing through the user’s digital identity. Hashing and cryptographic services, provided by HwSaaS and CryptaaS, support all the Blockchain transactions and enable the execution of zero-knowledge proof transactions on the Blockchain network. Multifactor authentication, enhanced with facial biometric authentication, leverages cryptographic services, especially for mission-critical services. The authentication tokens support IoT-enabled services to provide extensive authentication capabilities for the infrastructure, particularly enhanced with novel features such as secure distance bounding enabling proximal distance verification.

Within the Critical-Chains framework, the flow control of data pushed from the business applications on the Blockchain, or in general on any distributed cyber-physical layout is managed through a selective notification message system that sends the data into the injection components and enables the data to be pushed into the streaming database. Such a data injection mechanism is implemented through a Semantic Triple store, and directly binds the Big Data processing facility through the streaming tools. The whole data architecture provides the foundation for data visualisation and is powered by search utilities, enabling strong reporting and analytics capabilities.

The FMaaS provides business applications with an engine to describe and design rules and workflows as well as the key component that pushes the data and messages to all the components within the Critical-Chains main framework. The proposed Critical-Chains Main framework is effectively used in the presented use case as it provides an X-as-a-Service eco-system capable of supporting IoT-enabled financial or insurance services with few modifications. section 3 presents more detailed and descriptive information about the validated use case in the insurance domain.

### 2.1 Authentication and Cryptographic back-end

The authentication and cryptographic back-end are composed of the XaaSs, namely AuthaaS, CryptaaS, and HwSaaS, and the authentication token, namely the SecureStick. CryptaaS is a high-level software service which enables the basic functionalities of a typical Hardware Security Module (HSM, HwSaaS in Critical-Chains). HwSaaS is a physical device, a typical hardware security module, that is capable of carrying out major cryptography operations such as true random number generation, prime number generation, key generation and management, secure key storage and exchange, symmetric encryption (AES, 3DES), asymmetric encryption (RSA, ECDSA), and hashing (SHA) at FPGA level. HwSaaS is enclosed in a tamper-proof enclosure and operates on the server side. The low-level API of the HSM forms the HwSaaS which enables a micro-service type of usage (aligned with the XaaS model). HwSaaS and CryptaaS are highly interrelated as CryptaaS provides a software-level API (high-level at software components level) for the use of HwSaaS both of which enable the effective use of cryptographic functions by software developers e.g., Public Key Cryptography Standards 11 (PKCS11). CryptaaS provides the software-level integration of cryptographic functions, is fully compliant with the PKCS11 standards, and assists the BCaaS and AuthaaS in the following ways. First, CryptaaS enables the fast encryption of any financial transaction including blockchain transactions. Thus, CryptaaS behaves as a supplementary tool to enable encryption before injecting any data into the blockchain. Second, CryptaaS generates true random numbers and private keys (in cooperation with HwSaaS) which are used by AuthaaS. CryptaaS is also linked with the Enterprise Service Bus which is indispensable for the orchestration of all XaaS components.

The AuthaaS component consists of two modules: I) The authentication module which includes all sub-systems for user registration on the Critical-Chains platform and user authentication to access the platform, offering multiple authentication factors: login/password, attribute-based, and biometric authentication. To provide identity federation, the authentication module relies on standards and technologies, such as SAML and OpenID Connect, which simplify federated authentication and authorisation processes. Moreover, the module enables authentication of users with an eIDAS-compliant external identity provider, the Italian SPID (Public System for Digital Identity); II) The access control module includes common sub-systems for authorisation and secure access employing access tokens since user authentication is based on token-based authentication protocols.

SecureStick is the hardware component of AuthaaS which is a typical authentication token. It is designed for both person and node (or thing) authentication of registered users (authentication by something you have). SecureStick enables the Bluetooth Low Energy (BLE) chip with secure distance bounding features for the authentication of nodes (e.g. smartphone distance bounding). The HwSaaS and SecureStick are the twin hardware-based components supporting the protection of any transaction which uses the BCaaS and improving the resilience of the Secure Cyber Framework that protects the Critical-Chains main framework against cyber-physical attacks. The authentication needs of BCaaS are met by AuthaaS through multifactor authentication where the SecureStick is one of the three-factor authentications (the other two are password-based and facial biometric). The Secure Cyber Framework is designed to detect authentication-related cyber-attacks and potentially private data leakages by using the login and encryption history recorded by the Keycloak Authentication Service and is integrated with the records of AuthaaS (e.g. log file) for anomaly detection and recovery.

### 2.2 Secure distance bounding protocol and IoT integration

Nowadays, many IoT applications rely on secure location and proximity information, for example, contactless payment, entry systems without a physical key, or wireless access control. In these systems, the proximity between two entities is controlled by using wireless technologies. Even though most wireless systems have a limited communication range, relay attacks are a concern and pose a serious threat to wireless systems. A relay attack is an attack when a non-legitimate entity attempts to gain access by simply relaying the data between two legitimate entities. The attacker does not need to know the actual data being transmitted and is thus not stopped by encrypting the data which is transmitted between the two entities. To effectively mitigate relay attacks, authentication *per se* does not provide a complete safeguard; for this, it is necessary to add a secure distance bounding protocol ^30, 31^ to authenticate physically close to the system. The proposed passive secure-ranging protocol for Bluetooth Low Energy (BLE) radios involves two entities which are typically denoted as a verifier (e.g. car, person, etc.) and prover (e.g. keyfob or phone). The verifier controls access to a resource and the prover has to satisfy a proximity verification condition to gain access to the resource controlled by the verifier. As such, the secure distance bounding protocol is based on secure time-of-flight (ToF).

**Figure 2.**
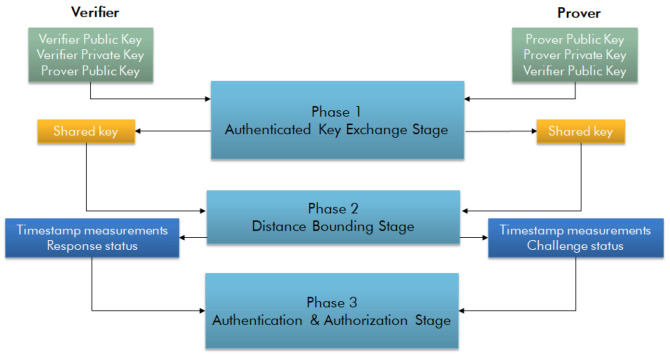
Secure distance bounding protocol.

As depicted in Figure 2, the SDB protocol has three stages: the authenticated key exchange stage, the distance bounding stage, and the authentication and authorisation stage. In the authentication key exchange stage, the communicating parties employ an authenticated key exchange protocol, the SIGMA-protocol is used, to generate a shared secret session key. In the distance bounding stage, the secure ranging is carried out based on the combination of timestamps, and security codes. During this stage, the verifier sends out challenges to the prover, and the prover responds directly to these challenges one by one. The following steps are repeated N times:

Step-1: The verifier sends a challenge to the prover. When sending the challenge, the verifier records the time of departure of the packet. The packet has a pseudo-random key which will change for every challenge. The pseudorandom key will be the access address of the packet.

Step-2: The prover receives the challenge and correlates on the access address. A right correlation to the access address means it is the expected packet and it is the moment to record the time of arrival. After the packet is completely received the prover will send a response which has a new access address, and a pseudo-random key generated using the session key. The prover also records the time of departure of the response.

Step-3: The verifier receives the response; the verifier correlates the address given to the access address. If the correlation is right, the time of arrival is recorded.

In the authentication and authorisation stage, the prover sends all estimated time-of-arrival and time-of-departure values and the status (True or False) of the received access addresses in the challenge packets to the verifier. The data is encrypted using the session key. Based on the received information from the prover and the information available for the verifier, the verifier makes an authentication decision and authorises the prover to access the requested resources if the authentication is successful.

The Critical-Chains main framework utilises LinkSmart which is an open-source IoT middleware delivered as a result of a previous EU project, namely Hydra ^32^. On the client side, the Linksmart IoT Device Gateway (DGW) is integrated with the user’s computer (portable device, tablet, smartphone, etc.) to collect data from the IoT device. The DGW operates as a data acquisition node which triggers the SecureStick (with an SDB feature) to collect data according to DGW configurations (in JSON format). On the other hand, the MQTT broker URL is defined in the device gateway configuration file. By following the configurations, the DGW publishes the related IoT information (i.e., proximal distance data, timestamp, farmer’s ID, wallet ID) to the defined MQTT topic. Note that the sensitive data are encrypted within the script which reads the SecureStick. The streaming data is available for both real-time data monitoring and historical data storing (to a database).

On the server side, the Linksmart Historical Datastore (HDS) component is used to store the data in a database. The HDS runs on the server (hosting the Critical-Chains Main Framework) which handles the database operations according to the predefined criteria. For instance, unlike DGW, the HDS configures only the parameters related to the database, (SQLite3 in our case), and Rest API. The data sources are defined by using the “registry” POST API, where the data source name, type (e.g., MQTT in our case), MQTT topic to subscribe, and Quality-of-Service (QoS) are sent via an HTTP POST request. Thus, every reading from the DGW is stored in the HDS. Finally, by using the HDS rest APIs, custom time-series queries can be applied to the historical data (i.e. by selecting all sensor data stored in a given period). Additionally, a Spring Boot REST API is developed for running high-level semantic queries and inferencing over previous custom queries which are not supported by HDS. Finally, the decryption is handled within the POST request. For example, the above-mentioned Spring Boot API enables the insurance company to query whether the user was at home for a given time interval, and what the percentage was of the farmer’s time spent in their quarantined area, i.e., their home.

### 2.3 True random numbers improving the Hardware-based Cyber Resilience

Critical-Chains innovation has strived to surpass the state-of-the-art. Researchers at Partner organisation ERARGE have achieved improvements in true random number generation (TRNG) that relies on the ring oscillator (see 25 and 26) and chaotic oscillator-based techniques (see 23 and 24) combined with corresponding vulnerability analysis (see 23 and 24). These TRNG designs have been applied for the HwSaaS as two options. Ring oscillator-based design can easily be implemented at the FPGA level without the need for extra hardware components. Moreover, this can result in high throughput (e.g., up to a few hundred MBits per second). The other design that utilises the chaotic oscillator, presents at least twice the throughput as compared to the ring-oscillator-based technique. The main drawback of the chaotic oscillator-based technique is that it requires additional hardware components to be implemented. This makes the latter approach more complex and expensive. These techniques have been applied for the HwSaaS which makes the underlying HSM resilient against attacks. The integrated approach that facilitates the two versions of SecureStick and the advanced HSM will push beyond the state-of-the-art as this approach will also be combined with a blockchain infrastructure (BCaaS), Cryptographic Services (CryptaaS, HwSaaS), and AI-enabled Secure Cyber framework. Moreover, the SecureStick has been implemented at the hardware level and integrated with the HSM at the laboratory scale, thus providing Proof-of-Concept. Subsequently, further enhancements of SecureStick to operate with Bluetooth Low Energy and ranging features that enable its wider integration with IoT.

### 2.4 Blockchain-as-a-Service

The proposed BCaaS includes the integration of well-known distributed ledger/blockchain technology: Quorum ^33^, and the Keyless Signature Infrastructure (KSI) Blockchain ^34^. The Quorum and KSI Blockchain technologies each provide essential integrity-checking services for the BCaaS. Quorum is responsible for implementing and maintaining the Ethereum-based blockchain; whereas KSI Blockchain is used to sign and secure the outputs of the transactions (financial or insurance) taking place over the network. KSI Blockchain can be used to sign and secure the data-hash roots produced by insurance transactions taking place over the network (for auditing purposes, for example). KSI Blockchain presents a globally distributed network infrastructure for providing cryptographically-secure signatures for any digital data set. KSI Signatures are independently verifiable proofs of integrity, signing time, and signing entity which are crucial information in any insurance claim verification settlement. KSI Blockchain makes use of cryptographic one-way hash functions (such as SHA-256) to transform data into a non-reversible, fixed-size hash value. This represents a digital fingerprint of the data that is collected by the IoT nodes, e.g., proximity data collected by the SecureStick.

Complementarily, Quorum provides a permissioned implementation of Ethereum which supports transactions and contract privacy. Quorum assures only authorized parties are given access to the platform network, Critical-Chains main framework in our case. Thus, Quorum enables a permissioned chain of people (i.e., farmers) in the system where data exchanges take place between participants who are pre-approved by a designated authority. Additionally, Quorum differentiates between public and private transactions. Open transactions are similar to those taking place on the Ethereum platform; whereas, private transactions are confidential, such as privacy-sensitive data like health status in case of the pandemic scenario for crop insurance.

## 3 The pandemic use-case for crop insurance

The COVID-19 crisis has affected the world in an unprecedented way. In addition to the public health effects of the disease, measures to contain the spread of COVID-19 have posed significant risks to the food sector through disruptions to food production, distribution, and access. The growth rates have significantly decreased, many farm workers have lost their jobs, and many farmers have stopped their production. For instance, a reduction in workforce availability due to COVID-19 is estimated to have reduced U.S. agricultural output by about USD 309 million in the period from March 2020-2021.

The main problems that were exacerbated during COVID-19 related to manpower supply, market access, lack of technology for inclusivity and resilience, and food security ^35^.

During the first wave of the pandemic European farmers suffered significant economic losses as a result of supply chain disruptions and/or the closure of specific trade channels (e.g., food service sector). The value of the agricultural industrial outputs declined by 1.4% in 2020 compared to 2019. Incomes significantly declined by about 8%. Manpower shortages became a serious problem because of lockdowns and travel restrictions. Among ornamental products, the horticultural category experienced significant financial losses due to COVID-19. As a result of this unexpected situation, farmers were faced with business interruptions and even company closures ^36^.

The COVID-19 pandemic and the measures taken to limit the spread of the disease have significantly disrupted economic activity in countries around the world. The insurance sector has helped farmers to mitigate their losses. Insurers provided many services by adapting their policies for health and life insurance, workers’ compensation, sick leave, indemnities and business interruption. The Association of British Insurers (ABI), estimated that they would pay GBP 900 million pounds for business interruption claims as of April 2020 ^37^. However, the majority of the farming industry, especially the small enterprises are still uninsured and not resilient to new lockdowns. Therefore, there is a need for claim verification even during lockdowns and other restrictions. Accordingly, the insurance sector needs more accountable and trustworthy technology-enhanced solutions to verify loss claims.

For those with relevant insurance policies, the effects of business interruptions and discontinuity in production processes could be mitigated through insurance companies compensating for the economic loss to some extent. There exist many reasons behind such interruptions such as lockdowns, travel restrictions, supply chain, and logistic problems. In many countries, if someone was infected by COVID-19, a quarantine procedure was applied for a certain period of time. In the case of wider spreads of COVID-19, farmers and farm workers could be quarantined in their homes for a period of weeks. Insurance companies have responsively introduced new policies and revised some of theirs for new clients. Understandably, the insurance sector is concerned with an accurate assessment of their liabilities by performing correct loss calculation and mitigation cost estimation cases arising from the pandemic and the impact of measures taken to counter it. Whatever the policy, insurance companies need to know that the affected farmers or farm personnel were/are COVID-19-positive, complying with the quarantine rules and not leaving their homes. Therefore, there is a strong need to develop quantifiable and trusted measures for farmers’ proximal location presence verification.

**Figure 3.**
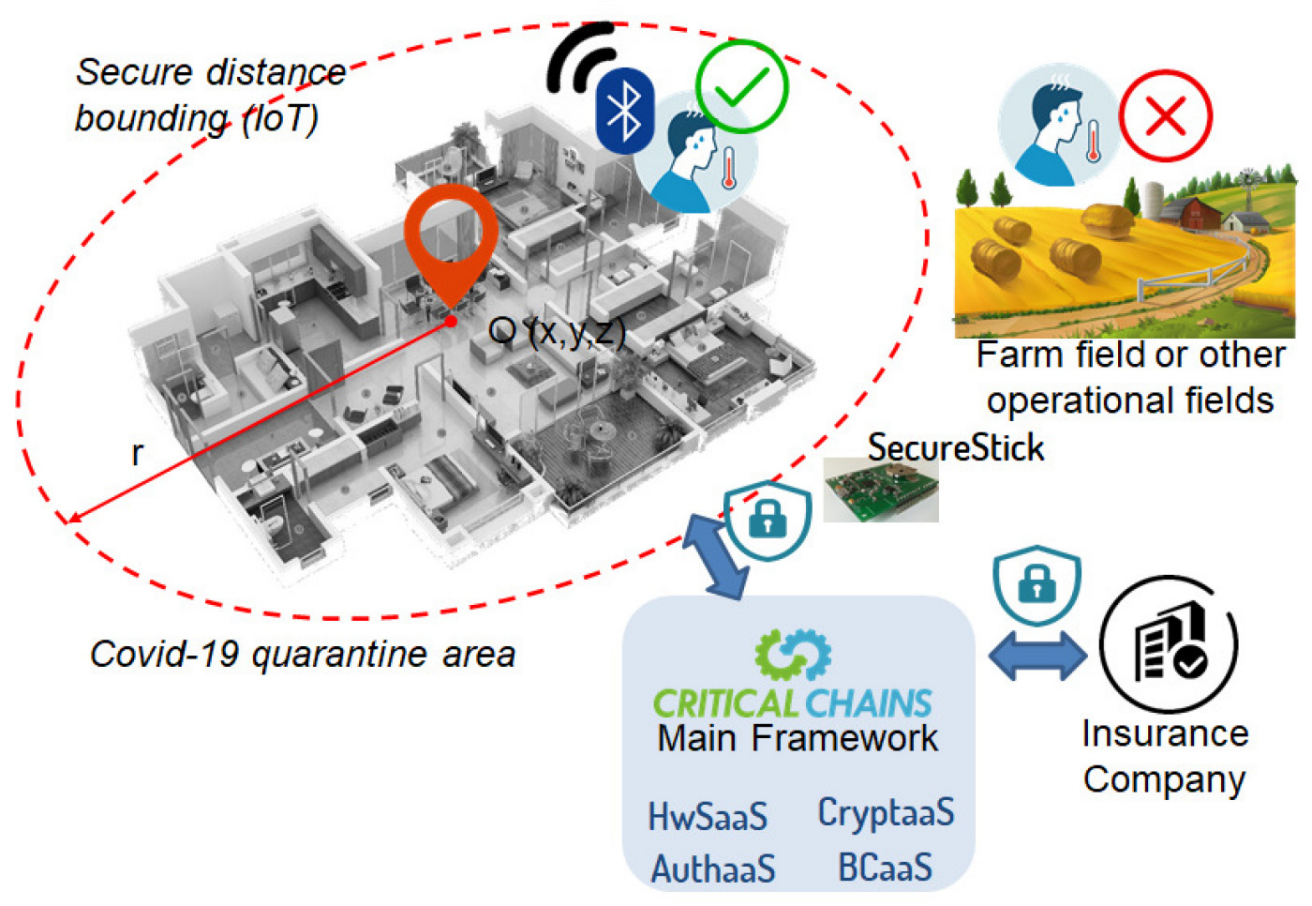
The proposed use-case concept.

AuthaaS plays a crucial role here in verifying the farmer themself as well as their SecureStick itself (i.e., node authentication). AuthaaS verifies that the farmer is staying at home during his/her quarantine period. Here, node authentication is realised by proximal location presence verification of the farmers. For instance, the technology can be applied as a wearable IoT device or a portable device that can be carried by the user. Moreover, through CryptaaS and HwSaaS, the Critical-Chains main framework supports the insurance claim verification process by linking the insurance company services with the end-user, the farmer in our case, guaranteeing a trusted end-to-end secure channel. See Figure 3 for the conceptual overview of the use case.

### 3.1 Architectural overview of the use-case

The solution concept is based on the effective use of the Critical-Chains main framework and its underlying services aiming to verify that the farmer is staying at home during their quarantine period. The main framework has to link the policy-holder (in this case the framer); the insurance company claims settlement department as the end-user is responsible for managing the insurance claim verification process, guaranteeing a trusted end-to-end secure channel. AuthaaS is the main service of the Critical-Chains framework which enables both person and node authentication. Here, node authentication is realised for the proximal location presence verification of the farmer. For instance, the technology can be applied as a wearable IoT device or a portable device that can be carried by a patient who is supposed to be under quarantine. HwSaaS and CryptaaS are complementary services of the Critical-Chains framework as these two components secure the insurance claim data, including the instantly monitored location data, and other personal data to be protected against both security and privacy threats. Finally, BCaaS works at the back end to enable data integrity and accountability which has been addressed in new-generation decentralised insurance services based on distributed ledgers and smart contracts.

**Figure 4.**
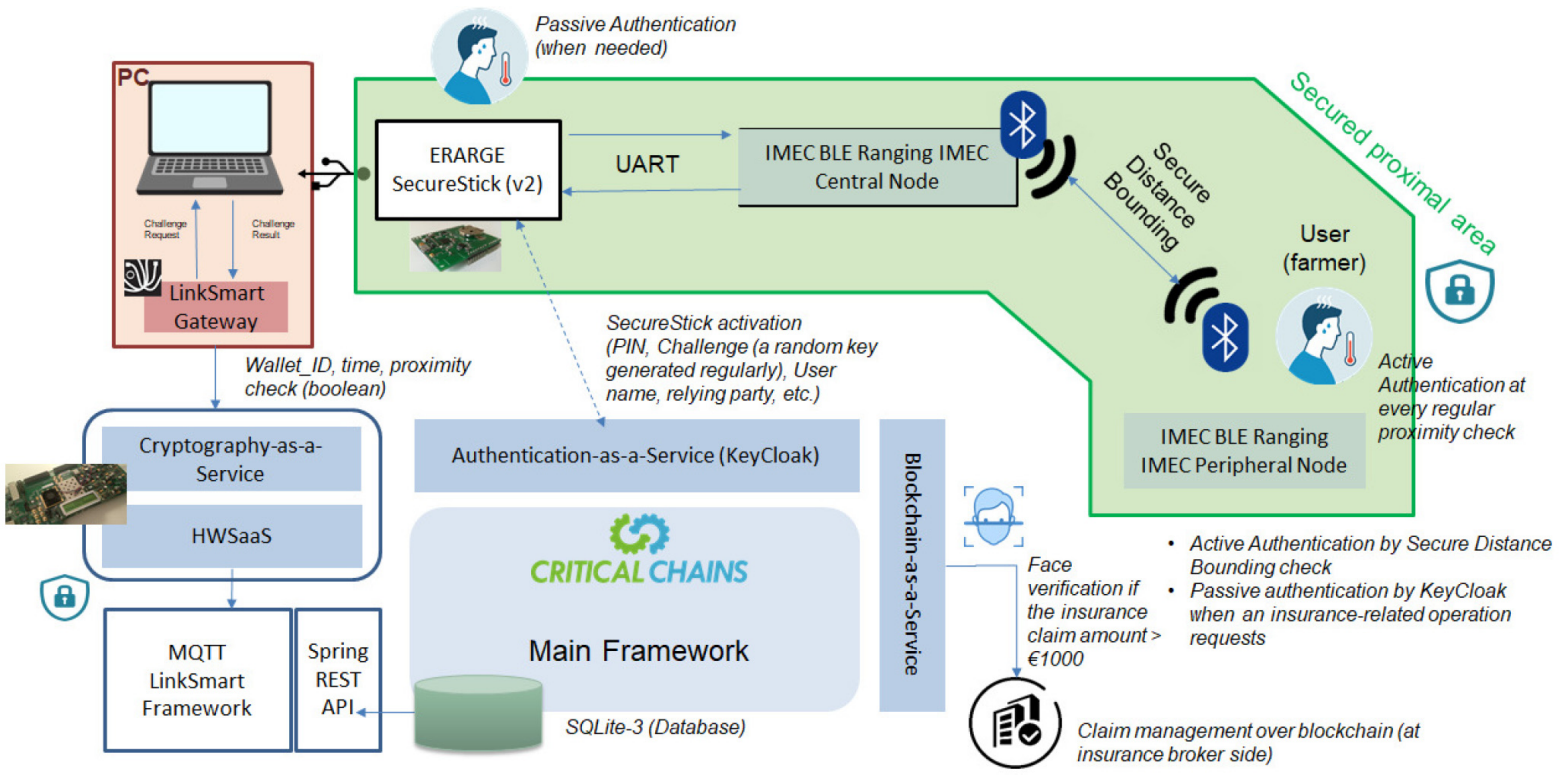
The proposed use-case system architecture.

Figure 4 presents an overview of the proposed solution architecture. The green area is the secured proximal area where the secure distance bounding is applied. There exists a peripheral node having BLE ranging capability and a central node installed in an appropriate location at home. The peripheral node is carried by the farmer and there is an active authentication mechanism that checks the proximal location presence regularly. The SecureStick is integrated with the BLE ranging central node and mounted on the PC. This PC delivers an IoT-enabled LinkSmart gateway. The gateway propagates the wallet ID, time, and proximity location data to the Critical-Chains main framework after encrypting the location and insurance claim data for security and privacy protection. The insurance claim data and the location presence information are stored on a secure database which is implemented by SQLite-3. The LinkSmart and its underlying publisher-subscriber solution, namely MQTT, are integrated with CryptaaS and HwSaaS and also the main framework through a Spring REST API. The proposed scheme also enables passive authentication by regular token-based authentication. This is applied when the quarantined user needs to access the main framework. Depending on the amount of challenge, say an insurance-related transaction, face verification can also be applied as an additional authentication mechanism for person verification. Facial recognition is only applied for higher-valued transactions; for instance when the insurance claim is higher than EUR 1000. BCaaS is used for distributed and decentralised claim management over blockchain by insurance brokers.

### 3.2 AuthaaS and SecureStick Evaluation

On the user’s side (client), SecureStick is used to authenticate a person using a front-end application. A front-end application runs on the user’s computer or smartphone. At the back end, the CryptaaS and HWSaaS run in close coordination. The authentication protocol relies on a multifactor authentication which ensembles the traditional user name and password which are supposed to be entered by the user, a one-time password that enables a more dynamic authentication mechanism, and facial verification as the biometric authentication (for more critical operations). SecureStick is developed by a two-step integration. First, the distance bounding and ranging applications are integrated with the authentication token at the hardware level. Second, the logical integration of SecureStick with HwSaaS is realised at a high level. Such a two-tiered integration strategy results in a more secure authentication token featured with distance bounding that can be used for both person and node authentication.

SecureStick also enables biometric authentication. To comply with the EU General Data Protection Rule (GDPR) and its national counterparts, facial biometric matching is realised at the device level. The so-called match-on-device applies the matching operation on the SecureStick itself which is implemented only on the embedded device owned by the user. The Convolutional Neural Network (CNN) is utilised for the detection and recognition of faces. It is widely adopted and performs well in particular for frontal faces ^38^. Since our use-case does not tackle highly-oriented faces (30 degrees or higher) and image resolution are not crucial (as the used web cameras provide sufficient quality), the achieved error rates seem promising for real-life applications.

The developed face authentication application is based on a lightweight, fast, and accurate 68-point landmark detector. The technique is based on CNN which presents satisfactory results. For face detection, a simple Single Shot Multibox Detector (SSD) is used although we are mainly dealing with single faces captured via the web cameras. The face detection model has been trained on the WIDERFACE dataset which is an open data set and widely preferred in many studies. The average delay time needed to detect a face is measured as 30 ms within a 38 fps video stream ^39^.

After detecting a face in a frontal image, the face authenticator computes 68-Point face landmarks for each detected face. The default model has a size of only 350kB (face_landmark_68_model) and the tiny model is only 80kB (face_landmark_68_tiny_mod). Both models employ the ideas of depth-wise separable convolutions as well as densely connected blocks. The models have been trained on a dataset of 35k face images labelled with 68 face landmark points. To perform face recognition, a face matcher is used to compare reference face descriptors to query face descriptors by applying Euclidean distance. The matching is held on the SecureStick to comply with GDPR. A ResNet-34-like architecture is implemented to compute a face descriptor for which we re-use the pre-trained models ^38^.

The developed face recogniser has been tested in an open facial image data set published by the Georgia Institute of Technology, USA ^40^. The data set contains images of 50 people taken in two or three sessions between 06/01/99 and 11/15/99 at the Centre for Signal and Image Processing at Georgia Institute of Technology. All people in the database are represented by 15 colour JPEG images with a cluttered background taken at a resolution of 640x480 pixels. The average size of the faces in these images is 150x150 pixels. The pictures show frontal and/or tilted faces with different facial expressions, lighting conditions and scales. Each image is manually labelled to determine the position of the face in the image. Five images with indexes starting from sixth to tenth for each subject are used to extract descriptors for each subject. These images are selected because they present a reasonable and realistic pose of a subject that may occur for a typical online banking application. They have been used to test whether the original subject is recognised or not. The imposter tests are conducted in two ways: I) Harsh case: The labelled facial feature is used but only the imposter subject’s descriptor is removed from the array. The Euclidean Distant between the enrolled and the queried samples are stored. This test includes all cross-checks with the rest of the subject pool. For instance, Subject#1 is compared with Subject#2 to Subject#50. II) Realistic Case: The labelled facial feature is used but only the target subject’s descriptor has been set for the cross-check. Here, the test subject is randomly selected from the rest of the subject pool. For instance, Subject#1 is compared with a randomly selected subject, say Subject#35 only. The results are given in terms of Equal Error Rate (EER) which is defined as the error rate where the false acceptance and false rejection rates are equal to each other. The EER obtained for the harsh case and the realistic case are reported as 0.95% and 0.44%, respectively.

As the results show, the recognition performance seems promising for the pilots and can be used effectively, especially for indoor applications. The node authentication performance is also measured. In this case, the authentication of the SecureStick (with SDB) is called by a web service within a 10-meter diameter area.

### 3.3 Secure distance bounding evaluation

**Figure 5.**
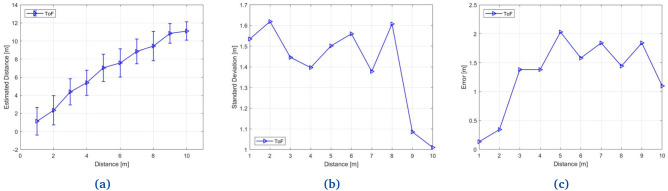
( **a**) Outdoor distance measurements results; ( **b**) The standard deviation of Time-of-Flight based distance measurements; ( **c**) Error in Time-of-Flight based distance measurements.

The verifier and prover are implemented on two NXP KW36 (BLE) SoCs to evaluate the Secure Distance Bounding. The evaluation is set up to take place in an outdoor environment. Each board is equipped with an omnidirectional antenna. The two nodes are placed a certain distance apart, the distance is varied (d = 1, 2, 3, .., 10) for each run. For each distance, the measurement is taken 250 times. Per distance measurement, 80 frequencies are used from the 2.4GHz ISM band to perform the distance measurement. The ToF distance estimation is based on the 80 frequency measurements. The results are shown in Figure 5a, where the precision of Time-of-Flight can be seen in a real and practical situation. The precision (i.e., the standard deviation) of the Time-of-Flight distance measurement is 1.6m, as can be seen in Figure 5b where we show the standard deviation of the Time-of-Flight measurements. Figure 5c shows the error in the distance measurements. This plot shows the maximum error of Time-of-Flight which is 2m.

Latency and energy consumption performance of the proposed SDB solution is other factors that are observed in this use case. Latency is a crucial factor as it is important to have the latest results as rapidly as possible. The latency is the time it takes to measure the distance based on Time-of-Flight and to compute the outcome of the measurements which results in a verification decision. On the other hand, the amount of energy gives an idea of what the added cost will be for the SDB, as this is important for IoT devices powered by a battery.

The Latency of one SDB procedure can easily be calculated. Since the number of measurements is set to 80, which means that 160 transfers will be made, for each transfer, one frame takes 400µs. The measurement period takes 64ms. The overall time from the start of the SDB process until a decision is made will be within 65ms. For the SDB process, we consider the power utilisation for the hardware execution as the key determinant of the power needed to realise the distance bounding. During the SDB process, the power utilisation in transmitting and receiving is dominant. The energy needed for one transmission is 5.7µJ and for one instance of receiving 7.4µJ. The energy needed for decision-making is approximately 17µJ. The energy per node for all the measurements is about 1.1mJ.

The resilience of the SDB is also considered in this study. The SDB is needed to prevent certain attacks such as impersonation attacks, relay attacks and early-detect and late-commit attacks. The impersonation attack is when a non-legitimate device attempts to be a legitimate prover. The relay attack is also called a man-in-the-middle attack. The man-in-the-middle is a non-legitimate device which attempts to relay the data of the verifier and the prover to get a positive decision from the verifier. The early-detect and late commit attacks are forms of relay attack, where the attacker detects the transmitted bit early and commits to its decision (whether the bit is a ‘1’ or a ‘0’) late.

The proposed solution enables a device to authenticate another device and securely determines its physical proximity. This SDB protocol combined with a Bluetooth LE radio gives the system designers the advantage of being secure, much less vulnerable to relay attacks and very power efficient compared to the other technologies available. Beyond Bluetooth security tokens, secure wireless distance bounding is particularly relevant for automotive secure access (keyless entry) and also secure building access applications.

### 3.4 Evaluation of the HwSaaS and CryptaaS

All cryptographic test procedures are carried out according to the PKCS11 standard. The cryptographic algorithm tests are classified into three main categories: i) Symmetric encryption algorithm tests; ii) Asymmetric encryption algorithm tests; iii) Hashing algorithm tests. The performance analysis of symmetric encryption algorithms, AES, DES, and 3DES, are presented in Table 1. As seen from the results, even for longer-bit algorithms, the reported speed is highly satisfactory and can be used for node authentication. Moreover, since symmetric algorithms present better resilience against quantum-based attacks in blockchain-based transaction environments, the new generation of Fintech and Insurtech services can use the proposed HwSaaS and CryptaaS.

**Table 1.** Performance of Symmetric Cryptography Algorithms.

Mode	Clock Cycle	Frequency	Speed	Frequency	Speed
AES-128	32	125 MHz	500 Mbit/s	250 MHz	1 Gbit/s
AES-192	38	125 MHz	420 Mbit/s	250 MHz	840 Mbit/s
AES-256	44	125 MHz	360 Mbit/s	250 MHz	720 Mbit/s
DES	17	125 MHz	470 Mbit/s	250 MHz	940 Mbit/s
3DES	17	125 MHz	450 Mbit/s	250 MHz	900 Mbit/s

In this study, asymmetric algorithms are also evaluated. RSA is the widely adopted algorithm which is used in many PKI systems. In many online finance and insurance services, asymmetric cryptography is used mainly for person authentication and encryption of financial or insurance-related transaction data. For 512-bit and 1024-bit RSA, 20 and 10 operation/s performance are achieved, respectively. This shows that when parallel HwSaaSs are used one can handle the operational needs of Fintech and Insurtech in real-life cases. Hashing also plays a critical role in blockchain-enabled frameworks, especially for the immutability of records and integrity checking. As presented in Table 2, SHA is applied for various bit lengths and very promising results are noted as even for high frequency and longer bits, one can achieve 3.6 Gbit/s. Such a speed is highly satisfactory for near-real-time services in IoT-enabled Fintech and Insurtech operations.

**Table 2.** Hashing Performance.

Mode	Clock Cycle	Frequency	Speed	Frequency	Speed
SHA1	73	125 MHz	897 Mbit/s	250 MHz	1.8 Gbit/s
SHA256	57	125 MHz	1.12 Gbit/s	250 MHz	2.24 Gbit/s
SHA512	73	125 MHz	1.8 Gbit/s	250 MHz	3.6 Gbit/s

The performance of CryptaaS and HwSaaS was also evaluated in terms of CPU load, memory utilisation, cryptographic latency, and throughput. The CPU Load refers to the amount of computational work that the CPU performs or has to perform. Memory utilisation, or memory usage, simply refers to the amount of memory that is currently being used. Cryptographic Latency is measured as the time needed to perform cryptographic operations whereas cryptographic throughput is the rate at which cryptographic operations can be performed.

For 1024 KB data samples, memory utilisation is approximately 3.9 MB and the average CPU load is 5.54% for the CryptaaS. The throughput is about 500 KB with an average latency of 1.8 ms. For HwSaaS, the memory utilisation is approximately 5 MB but with a much better CPU load of 2%. The throughput is higher as 1.8 Gbit/s is observed with significantly less latency of 0.4 ms. These results show that both software-based (CryptaaS) and hardware-based (HwSaaS) HSMs can be effectively used in Fintech and Insurtech IT infrastructures. Note that CryptaaS and HwSaaS are evaluated over an Intel(R) Core(TM) i7-3770 CPU @ 3.40GHz with 3837MB RAM at 2666 MHz. The HwSaaS is implemented on the Kintex-7 FPGA board with a fixed oscillator enabling differential 200MHz output, 1GB DDR3 RAM and 128MB linear flash memory for PCIe.

## 4 Conclusions

This paper has presented a detailed description of the architecture, development, and validation of a solution stack to deliver real-time hardware-enabled services comprising Authentication (including Distance Bounding and Prover’s time-limited Proximal Location Presence Verification) supported by Hardware Security and Cryptography (AuthaaS, HwSaaS, CryptaaS). This is delivered through the Critical-Chains Main Framework in which, the back-end server data integrity is assured through Blockchain-as-a-Service (BCaaS).

The system has been validated within the insurance claim settlement application domain, specifically to support the insurers in the forensic verification of statements relating to the insurance claims; this also entails the verification of the presence of the claimant at a particular place (e.g., home) during a particular period.

In terms of key performance criteria (latency, throughput, power consumption) and resilience against impersonation, tampering and relay attacks, the system performance has proved satisfactory. Specifically, the performance evaluation of the HwSaaS and CryptaaS based on the PCKS11 standard for including symmetric, asymmetric and hashing algorithms tests (including longer bit algorithms) have demonstrated satisfactory results. This includes key criteria such as CPU load, memory utilisation, cryptographic latency, and throughput indicating the system is scalable for operational deployment e.g., in Fintech and Insurtech. Moreover, as symmetric algorithms are more resilient against quantum-based attacks on the blockchain environment, this promises greater security for emergent Fintech and Insurtech services.

The validation of the proposed Secure Distance Bounding (SDB) solution also demonstrated satisfactory performance in terms of resilience, latency and power efficiency thus proving to be a scalable solution to protect against relay attacks. SDB is particularly useful given a wearable IoT device or a portable device that can be carried by the Prover who is to comply with a certain location-time place-ability stipulation which has to be verified, e.g., in application domains such as probation conditions compliance assurance, automotive secure access (keyless entry) and also secure building access applications. Thus, the Critical-Chains secure authentication and distance bounding has delivered a scalable trusted system solution for real-time secure authentication-as-a-service underpinned by hardware-enabled security, encryption and Blockchain-as-a-service (BCaaS).

## Data Availability

All data underlying the results are available as part of the article and no additional source data are required.
