# Supplementary material for: Integrated secure distance bounding and hardware-based security: A case study for the insurance claim verification of farmers during COVID-19
Source: Open Res Eur. 2023 Feb 23;3:40. [Version 1] doi: 10.12688/openreseurope.15448.1 (PMC11336557; doi:10.12688/openreseurope.15448.1)
Supplement: Peer review history

# Reviewer report: Reviewer response for version 1

**DOI:** 10.21956/openreseurope.16701.r30944

**Published:** 2024-08-20

**Authors:** Abdulhamit Subasi

**Affiliations:** 1 Institute of Biomedicine, Faculty of Medicine, University of Turku, Turku, Finland

This paper presents the practical application of IoT and hardware-based cyber security in the Fintech and Insurtech domains. The authors present sufficient information about the state-of-the-art covering the urgent needs of the insurance and finance sector. The paper is based on practical applications in a promising European project, called Critical-Chains, where the X-as-a-Service approach is clearly described.

 The proposed techniques, in general, rely on well-known methods. From this perspective, the paper does not present a significant scientific novelty. However, the overall solution architecture is worth to be followed by the academic and industry community because the proposed main framework can inspire other developers and can be seen as a good example of service-based integration of hardware and software solutions.

 The Secure Distance bounding (SDB) approach and the underlying protocol are good examples of secure proximity control and this has been improved with the crypto-as-a-service and authentication-as-a-service (node and person authentication). As a proof-of-concept application, the proposed SDB and IoT integration (enhanced with cyber security and blockchain services) is promising. However, there is a need to go deeper, especially in terms of performance verification, when the solution is decided to be promoted to the market and real-life applications.

 Although the authors mentioned Blockchain-as-a-Service seems not directly implemented in the sample use case (pandemic use case). Further clarifications and more technical details can be presented in a successor paper.

 The selected use case is a good example of presenting the technical capabilities of the proposed solution stack. However, the use case story behind this may not apply to all countries or can be obsolete after some time. It can be a good idea to add more information about the other use case options to open the mind of readers and help them correlate with their studies and create new cases of application.

 Overall, the paper is a good example of a use case paper presenting the concrete outputs of a European project and may help the industry and academia to link their studies with the IoT-enabled hardware-based security and blockchain needs of the insurance and finance sector.

 English language is acceptable in general, but there are some errors that should be corrected. You should very carefully check the language.

Is the rationale for developing the new method (or application) clearly explained?

Yes

Is the description of the method technically sound?

Yes

Are the conclusions about the method and its performance adequately supported by the findings presented in the article?

Yes

If any results are presented, are all the source data underlying the results available to ensure full reproducibility?

Partly

Are sufficient details provided to allow replication of the method development and its use by others?

Yes

Reviewer Expertise:

AI, Machine learning, Cybersecurity

I confirm that I have read this submission and believe that I have an appropriate level of expertise to confirm that it is of an acceptable scientific standard.

# Reviewer report: Reviewer response for version 1

**DOI:** 10.21956/openreseurope.16701.r32261

**Published:** 2023-07-21

**Authors:** Nikolaos Athanasios Anagnostopoulos

**Affiliations:** 1 University of Passau, Passau, Germany

The authors present a secure distance-bounding method within the framework of the Critical-Chains project. They try to motivate this method as a solution for insurance claims in the context of missing work due to illness, and other use cases, that may be dependent on distance bounding. In particular, they consider the verification of insurance claims for missed work days, made by farmers who may have had COVID-19.

 In general, this use case appears slightly artificial, and the presented solution would face privacy issues which have not been fully addressed, if applied to this scenario. Moreover, the authors suggest the utilisation of a wearable IoT device to ensure distance bounding. However, one could either remove this device, if it was not checking if it is currently being worn by a human, or one could have someone else wear it, e.g., a farmer could have his/her spouse wear this device instead of them. Facial biometrics identification seems to be suggested, and it seems to be suggested that this may be compliant with the EU General Data Protection Rule (GDPR), but it is not certain that the proposed solution would still be considered as privacy-friendly. It is not discussed how long the user may be supposed to wear the device, and how often biometrics-based authentication may be required. Generally speaking, a scenario regarding probation-term assurance in relation to a suspended prison sentence would have been easier to justify the use of the proposed system.

 The authors also fail to discuss the specific application of their system to the particular use case considered. For example, sentences such as "The authentication protocol relies on a multifactor authentication which ensembles the traditional user name and password which are supposed to be entered by the user, a one-time password that enables a more dynamic authentication mechanism, and facial verification as the biometric authentication (for more critical operations)." and "SecureStick also enables biometric authentication." are included, but it is never explained how biometric authentication is supposed to be applied to the use case of a farmer having been ill with COVID-19 and needing to prove he or she stayed home to successfully claim some insurance benefits. The authors really need to more adequately connect the individual parts of the solution they propose to the use case they are supposed to examine.

 Moreover, some statements, such as "For instance, a reduction in workforce availability due to COVID-19 is estimated to have reduced U.S. agricultural output by about USD 309 million in the period from March 2020-2021." really require a citation or some reference link. Additionally, the phrase "from March 2020-2021" makes no sense at all.

 The document has a rather large number of language issues, ranging from hyphens missing for compound adjectives, along with too many and too long compound adjectives e.g., "Prover’s Proximal Location Presence Verification" and "specific location-time bound prover’s presence verification", to random capitalisations, acronyms not being introduced in their first instance, etc. Moreover, the third affiliation is provided as "Stichting IMEC, Eindhoven, Netherlands Antilles", while Eindhoven is in the Netherlands, not in the Netherlands Antilles... Additionally, sometimes words forming acronyms are capitalised, and other times not. Furthermore, sometimes articles are missing. In Figure 1, "privilage" should have been "privilege". Sometimes, hyphenation should not be used, e.g., "thus providing Proof-of-Concept" should have been "thus providing a proof of the concept" or "thus providing a proof implementation of this concept". "The Euclidean Distant between..." should be "The Euclidean distances between..." The authors can access a commented version of their manuscript here, in order to address such language issues.

 Furthermore, some terms, such as "a Semantic Triple store", "eIDAS", etc., also need to be introduced and explained, perhaps in a footnote or through a citation referenced. The (REpresentational State Transfer) REST API ("API" should also be explain as an acronym) should also be somehow introduced (and capitalised) where it is being referred to. In the phrase "The so-called match-on-device applies the matching operation on the SecureStick itself", it is not clear what "match-on-device" refers to... On page 10, a tiny model is referred to in the phrase "and the tiny model is only 80kB (face_landmark_68_tiny_mod)", but it has not been referred to anywhere else... In the sentence "A ResNet-34-like architecture is implemented to compute a face descriptor for which we re-use the pre-trained models.", the meaning of "a face descriptor for which we re-use the pre-trained models" is not clear. "pilots" in the phrase "the recognition performance seems promising for the pilots" is rather hard to understand... "IMEC BLE" is referred to in Figure 4, but "IMEC" has never been introduced/explained...

 In 3.4, the evaluation system should rather be mentioned at the beginning and not at the end of the subsection. "The throughput is about 500 KB..." should rather be "The throughput is about 500 KB/s...". In "These results show that both software-based (CryptaaS) and hardware-based (HwSaaS) HSMs", HSM stands for Hardware Security Modules, thus a software-based HSM is rather paradoxical as a phrase; perhaps, "security modules" should be used instead of "HSMs". In "Intel(R) Core(TM)", the copyright and the trademark symbols should rather be used.

 For data availability, the statement: "All data underlying the results are available as part of the article and no additional source data are required." is rather not true. The article concerns an implementation and Figure 5 shows quantitative results, thus all the relevant data should have been provided... I failed to find where these data, e.g., the measurements used for Figure 5, may have been disclosed.

 Finally, the acronyms in the titles of the References need to be correctly capitalised. For Reference 16, the title should be "Blockchain and IoT in developing FinTech ecosystem – An assistance to insurance industry". References 31, 32, 33, 37, and 38, need the appropriate authors' names and titles. For example, Reference 31, instead of having "US020200264297A120200820" as a title, should have the appropriate U.S. patent number as a title, in the form of "U.S. Patent XXXXX", and the patent's applicants as authors... The reference link also does not seem to be the appropriate one, being https://www.netify.ai/resources/domains/storage.googleapis.com.

 In general, this work suffers from language issues and does not connect the proposed solution to the suggested use case very well. Moreover, the privacy issues of the proposed solutions are not adequately addressed in the context of the suggested use case: the solution may be GDPR-compliant, but an insurance company cannot really force the person insured to wear a proximity tracker all day long. In the case of a sentenced offender, the state may do so, but in the case of a farmer claiming insurance benefits the requirement for such invasive tracking is rather infringing on human rights, especially in relation to privacy. Thus, I cannot state that I consider this work as fully sound and valid.

Is the rationale for developing the new method (or application) clearly explained?

Yes

Is the description of the method technically sound?

Yes

Are the conclusions about the method and its performance adequately supported by the findings presented in the article?

Yes

If any results are presented, are all the source data underlying the results available to ensure full reproducibility?

No

Are sufficient details provided to allow replication of the method development and its use by others?

Yes

Reviewer Expertise:

Hardware security

I confirm that I have read this submission and believe that I have an appropriate level of expertise to confirm that it is of an acceptable scientific standard, however I have significant reservations, as outlined above.

# Reviewer report: Reviewer response for version 1

**DOI:** 10.21956/openreseurope.16701.r32262

**Published:** 2023-07-14

**Authors:** Milad Taleby Ahvanooey

**Affiliations:** 1 Nanyang Technological University, Singapore, Singapore

Thanks for your contributions to the area of multifactor authentication. After reviewing all the sections of the current version, I have decided to recommend a major revision as there are several technical shortages and questionable descriptions. Below, some constructive comments are summarized.

1. In the proposed motivating scenario, there is a lack of cyber attacks on the proposed architecture, raising the question of its efficiency in active cyber attacks. I suggest adding a separate section to consider the side-channel and network attacks during the R1 stage and providing an empirical analysis of the proposed schemes against such attacks.
2. In the literature, researchers introduced many MFA schemes partially similar to the proposed schemes in this article. Hence, it is necessary to include a comparative analysis section after the experimental evaluation to support your contributions and results. You need to compare your proposed schemes with at least three recent state-of-the-art MFA protocols.
3. In Figures 3 and 4, there are many lengthy labels that are larger than the icons. I would suggest reducing the size of labels to one-word or less lengthy phrases. Also, please include the cyber attacker in these scenarios as it is an inevitable fact through cyberspace.

Is the rationale for developing the new method (or application) clearly explained?

Partly

Is the description of the method technically sound?

Partly

Are the conclusions about the method and its performance adequately supported by the findings presented in the article?

Partly

If any results are presented, are all the source data underlying the results available to ensure full reproducibility?

Partly

Are sufficient details provided to allow replication of the method development and its use by others?

Partly

Reviewer Expertise:

Internet of Everything (IoE) Security, Authentication Schemes, Smartphone Security, Malware Analysis, Blockchain-based systems, and Federated learning.

I confirm that I have read this submission and believe that I have an appropriate level of expertise to state that I do not consider it to be of an acceptable scientific standard, for reasons outlined above.

## References

[1] : Blockchain-Based Lightweight Multifactor Authentication for Cell-Free in Ultra-Dense 6G-Based (6-CMAS) Cellular Network. *IEEE Access* .2023;11: 10.1109/ACCESS.2023.3249969 20524-20541 10.1109/ACCESS.2023.3249969

[2] : Efficient and Privacy-Preserving Blockchain-Based Multifactor Device Authentication Protocol for Cross-Domain IIoT. *IEEE Internet of Things Journal* .2022;9(22) : 10.1109/JIOT.2022.3176192 22501-22515 10.1109/JIOT.2022.3176192

[3] : Secure multi-factor access control mechanism for pairing blockchains. *Journal of Information Security and Applications* .2023;74: 10.1016/j.jisa.2023.103477 10.1016/j.jisa.2023.103477

[4] : Blockchain-based parking sharing service for smart city development. *Computers and Electrical Engineering* .2022;103: 10.1016/j.compeleceng.2022.108267 10.1016/j.compeleceng.2022.108267

# Reviewer report: Reviewer response for version 1

**DOI:** 10.21956/openreseurope.16701.r30946

**Published:** 2023-03-27

**Authors:** Baran Cürüklü

**Affiliations:** 1 School of Innovation, Design and Engineering, Mälardalen University, Västerås, Sweden

**Background and motivation:** Digitalisation pushes forward servitisation in the areas of fintech, insurtech, open banking, and mobile money business models, with the purpose of ensuring strong authentication. The need is related to the protection of security and privacy of data. This requires security-integrity of insurance and financial transactions.

**Summary of the work: **This work assumes a solution stack to deliver real-time hardware-enabled services comprising authentication supported by hardware security and cryptography; through the Critical-Chains Main Framework in which. The proposed solution stack system has been validated within the insurance claim settlement application domain. Thus, the proposed solution has been tested and validated in detail.

**Review summary:** This paper is based on EU-project, a fairly large consortium. The proposed work in this paper is sound. The contribution has been tested and validated in real-world applications, which also have critical importance, i.e. the contribution of this work is relevant. The language need no further review. The figures are clear, however, Fig 4 may need higher resolution and Fig 5 text could be larger.

Is the rationale for developing the new method (or application) clearly explained?

Yes

Is the description of the method technically sound?

Yes

Are the conclusions about the method and its performance adequately supported by the findings presented in the article?

Yes

If any results are presented, are all the source data underlying the results available to ensure full reproducibility?

Yes

Are sufficient details provided to allow replication of the method development and its use by others?

Yes

Reviewer Expertise:

Artificial inteligence (AI), AI-agent design, Cyber-physical systems

I confirm that I have read this submission and believe that I have an appropriate level of expertise to confirm that it is of an acceptable scientific standard.
